# Construction and properties of the silk fibroin and polypropylene composite biological mesh for abdominal incisional hernia repair

**DOI:** 10.3389/fbioe.2022.949917

**Published:** 2022-09-06

**Authors:** Fengming Luan, Wangbei Cao, Chunhui Cao, Baizhou Li, Xiaoyu Shi, Changyou Gao

**Affiliations:** ^1^ Department of Gastrointestinal Surgery, Second Affiliated Hospital of Zhejiang University, School of Medicine, Hangzhou, China; ^2^ Department of Polymer Science and Engineering, Zhejiang University, Hangzhou, China; ^3^ Department of Pathology, Second Affiliated Hospital of Zhejiang University, School of Medicine, Hangzhou, China

**Keywords:** silk fibroin, polypropylene mesh, abdominal incisional hernia, abdominal adhesion, hernia repair

## Abstract

**Background:** In this study, a new composite biological mesh named SFP was prepared by combining silk fibroin with polypropylene mesh. The mechanism and clinical application value of the SFP composite mesh were explored.

**Methods:** The fibrous membrane was prepared by electrospinning of silk fibroin. The silk fibrous membrane was adhered to the polypropylene mesh by fibrin hydrogel to make a new composite mesh. The characterizations were verified by structural analysis and *in vitro* cell experiments. A total of 40 Sprague–Dawley rats were randomly divided into two groups, and 20 rats in each group were implanted with the SFP mesh and pure polypropylene mesh, respectively. The rats were sacrificed in batches on the 3rd, 7th, 14th, and 90th days after surgery. The adhesion degree and adhesion area on the mesh surface were compared, and a histopathological examination was carried out.

**Results:** *In vitro* cell function experiments confirmed that the SFP mesh had good cell viability. The control group had different degrees of adhesion on the 3rd, 7th, 14th, and 90th days after surgery. However, there was almost no intraperitoneal adhesions on the 3rd and 7th days after surgery, and some rats only had mild adhesions on the 14th and 90th days after surgery in the SFP group. There were statistically significant differences in the postoperative intraperitoneal adhesion area and adhesion degree between the two groups (*p* < 0.05). Histopathological examination confirmed that the mesenchymal cells were well arranged and continuous, and there were more new capillaries and adipocyte proliferation under the mesenchymal cells in the SFP group.

**Conclusion:** The SFP mesh shows good biocompatibility and biofunction *in vitro* and *in vivo*. It can promote the growth of peritoneal mesenchymal cells. The formation of a new mesenchymal cell layer can effectively reduce the extent and scope of adhesion between the mesh and abdominal organs. The SFP mesh will have a good application prospect in the field of abdominal wall hernia repair.

## Introduction

Abdominal incisional hernia is one of the main complications of abdominal surgery due to the increased intra-abdominal pressure and protrusion of abdominal contents in the case of incomplete healing of abdominal incision muscularis and fascia (Skrobot et al., 2016). According to the report by Bosanquet and Steensel, the incidence of incisional hernia was 12.8% within 2 years after abdominal surgery (Bosanquet et al., 2015; van Steensel et al., 2020) and 69% in high-risk patients (Alnassar et al., 2012). The risk of developing a hernia is five times higher if the incision infection occurs after surgery, combined with factors that can lead to fascial defects, including smoking, obesity, malnutrition, and steroid use (Bensley et al., 2013). Abdominal incisional hernia can lead to respiratory insufficiency and circulatory system dysfunction and even abdominal organ injury, such as intestinal obstruction and intestinal rupture, as well as a negative impact on the stability of the spine and thorax. The quality of life of patients is seriously affected (van Ramshorst et al., 2012; Paulo et al., 2020; Sahin et al., 2020; Shao et al., 2020; Alayon-Rosario et al., 2021; Hoffman et al., 2021; Jensen et al., 2021; Licari et al., 2021; Soare et al., 2021; Cassese et al., 2022).

Surgical mesh repair is still the main clinical treatment for incisional hernia of the abdominal wall (Yang et al., 2020a; Hoffmann et al., 2021). The general treatment strategy for incisional hernia is as follows: small incisional hernias (hernia ring diameter<5 cm) can be repaired with suture alone. However, for moderate or higher incisional hernias (hernia ring diameter>5 cm), reinforcement repair with repair materials should be adopted. Although some risk factors can be avoided, there is still a high possibility of recurrent hernia after surgery with simple incisional hernia suture repair without mesh material. It often makes further clinical treatment difficult. According to reports, the recurrence rate of incision hernia without repair material can be as high as 51% (Hesselink et al., 1993), while the recurrence rate of incisional hernia with repair materials can be reduced to 10%–24% (Luijendijk et al., 2000).

The traditional mesh can be divided into three types according to the materials: 1) single component non-absorbent material mesh such as polypropylene, polyester, and polytetrafluoroethylene; 2) composite material mesh such as Johnson’s Proceed mesh (polypropylene, polydioxane, and oxidized regenerated cellulose) and Barder’s Composix mesh (polypropylene and polytetrafluoroethylene); 3) biological mesh such as the SIS mesh (porcine small intestine submucosa) by Cook Company, etc. Although the use of mesh greatly reduces the recurrence rate of incisional hernia and promotes wound healing, according to a large number of clinical data feedback, the polyester mesh is rarely used due to poor anti-infection ability and heavy foreign body reaction. The polypropylene mesh has good histocompatibility, light inflammatory response, and strong anti-infection ability, but it is easy to adhere to viscera, resulting in intestinal obstruction, intestinal leakage, and other complications (Aydemir Sezer et al., 2019; Yang et al., 2020b; Nessel et al., 2021; Pascual et al., 2022). Expanded polytetrafluoroethylene (ePTFE) is not easy to adhere to viscera, but it has poor anti-infection ability and poor firmness. To overcome the aforementioned shortcomings, polypropylene/expanded polytetrafluoroethylene composite mesh and light polypropylene mesh were produced, but the actual clinical effect is not particularly ideal. The ideal hernia mesh should have the following characteristics: 1) high tensile strength; 2) monofilament structure to avoid bacteria hiding; 3) tardiness for infection; 4) good biocompatibility so that fibroblasts can be induced to grow into the mesh pores and form a “reinforced cement-like” structure; and 5) less or non-degradation, inflammation, and foreign body reaction *in vivo*. The existing mesh materials in the market do not meet the requirements of “ideal mesh,” and it is urgent to develop new mesh to meet the needs of patients.

As a natural polymer material with good biocompatibility, silk fibroin has been widely used in biomedical fields, including ophthalmic surgical sutures, artificial corneas, artificial tendons, and ligaments in orthopedics, cartilage engineering, artificial skin on the wound surface in the trauma department, and anticoagulant scaffold in the cardiology department (Liu et al., 2012; Jeong et al., 2014; Nakayama et al., 2020). China is a big silk producer with abundant silk fibroin and good basic scientific research on silk fibroin. The protein content of silk is up to 98 percent, consisting of 70–80 percent silk fibroin and 20 to 30 percent sericin covered on the outside (Zhou et al., 2000). As a new biomedical material, silk fibroin has the following advantages: 1) good biocompatibility with negative charge; 2) the ability to promote cell adhesion. The silk fibroin has a mixed structure of a crystalline zone and an amorphous zone, and there are many basic amino acids in the amorphous zone, which has a certain adsorption effect on cells. Experiments have proved that silk fibroin can effectively adsorb peritoneal mesenchymal cells. Peritoneal mesenchymal cells can produce hepatocyte growth factor (HGF), which stimulate the proliferation and migration of mesenchymal cells, and express transforming growth factor-β antibodies. By promoting the secretion of T-PA, inhibiting the secretion of PAI-L, and improving peritoneal fibrinolysis, the occurrence of postoperative abdominal adhesion can be reduced. 3) It can promote the formation of neovascularization and has a certain anti-infection effect (Alnassar et al., 2012); 4) it degrades slowly in the human body; 5) mesh can be processed into membrane, particle, fiber, bracket, and other forms, and the processing process is relatively simple (Altman et al., 2003; Wang et al., 2008; Martinez-Mora et al., 2012). Based on the aforementioned characteristics, we believe that silk fibroin is a suitable material for making composite mesh.

**FIGURE 1:**
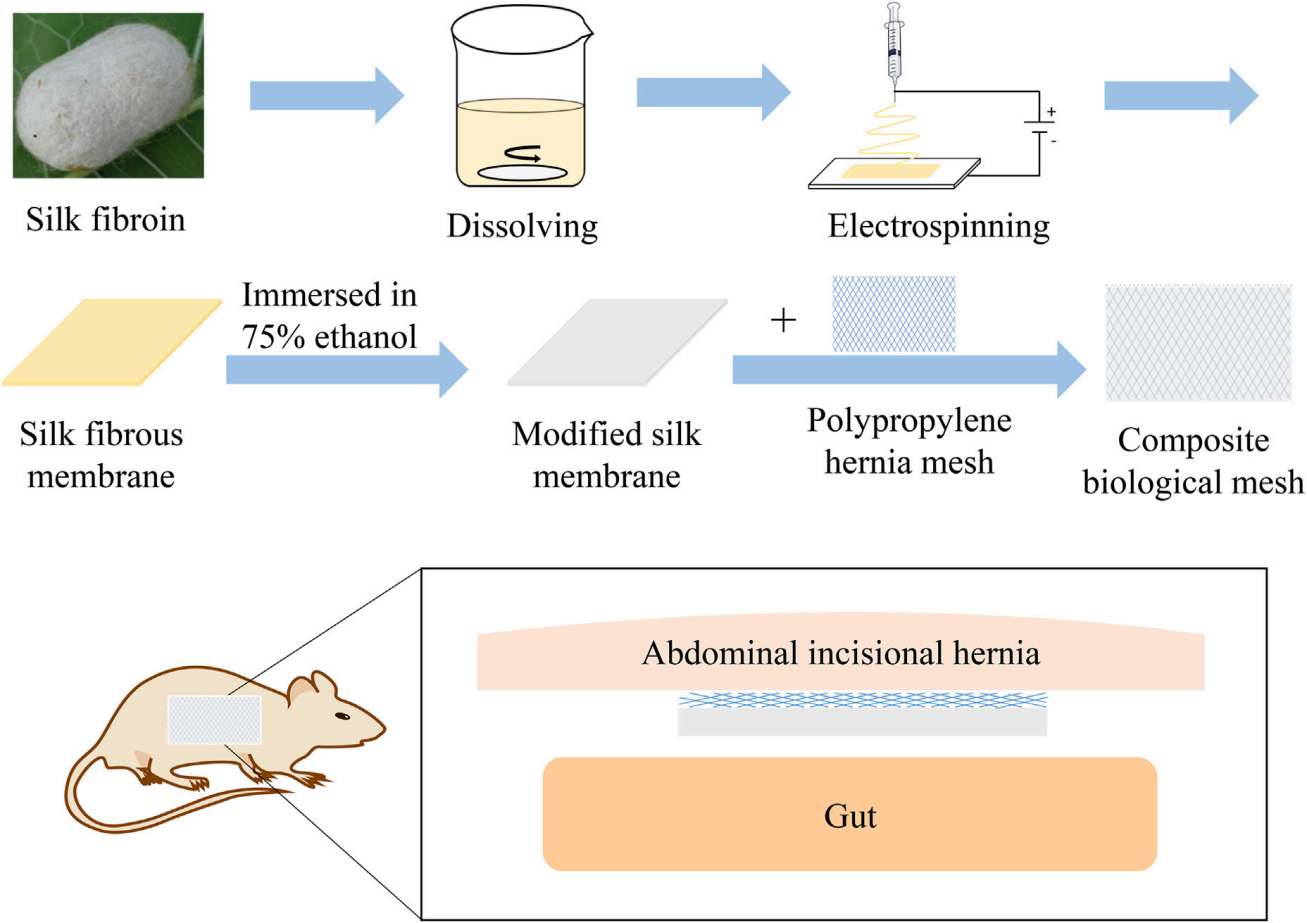
Schematic illustration of the silk fibroin and polypropylene composite biological mesh for abdominal incisional hernia repair.

In our study, combining the advantages of the previous polypropylene hernia mesh, the composite biological mesh of silk fibroin and polypropylene was developed for abdominal incisional hernia repair. The fibrous membrane is prepared by electrospinning of silk fibroin. The silk fibrous membrane is modified by soaking in 75% ethanol and then is adhered to the polypropylene mesh by fibrin hydrogel to make a new composite biological mesh. The advantages of the macroporous polypropylene mesh, such as good tensile strength, easy tissue growth, and being light and soft, are combined with the characteristics of silk fibroin, such as easy degradation, anti-adhesion, and promoting cell adsorption and angiogenesis. The composite biological mesh with good biocompatibility of silk fibroin is thus obtained. Experiments *in vitro* and *in vivo* were conducted to verify the characterization and clinical practicability of the SFP mesh (Figure 1), providing a new material and theoretical basis for the treatment of abdominal incisional hernias.

## Materials and methods

### Materials

Silk fibroin (SF) was purchased from Suzhou Simeite Inc., China. Ethanol and 1,1,1,3,3,3-hexafluoroisopropanol were purchased from Aladdin Biochemical Technology Co., Ltd., China. Thrombin and fibrinogen were purchased from Harbin Hanbang Medical Science and Technology Co., Ltd, China. Dulbecco’s modified Eagle’s medium (DMEM) (containing 100 μg/ml streptomycin and 100 U/mL penicillin) was purchased from Gibco, Thermo Fisher Scientific, Inc., United States. Fetal bovine serum (FBS) was purchased from Sijiqing Inc., China. Calcein AM and propidium iodide (PI) were purchased from Thermo Fisher Scientific Co., Ltd., United States. The polypropylene mesh was purchased from Beijing TransEasy Medical Technology Co., Ltd. The water used in this work was purified using a Millipore Milli-Q purification system.

### Mesh making

In total, 4 g of silk fibroin was dissolved in 40 ml of 1,1,1,3,3,3-hexafluoroisopropanol, which was added into a syringe. The syringe was placed in the electrospinning machine (Tongli micro nano manufacturer, TL-Pro), and the extrusion speed was adjusted to 4 ml/h. The voltage of the syringe nozzle was set to +15 kV. The drum covered with the release paper was used to receive spinning fibers. The distance from the drum to the nozzle was set at 15 cm. The voltage of the drum was set at −8 kV. The speed of the drum was set at 300 rpm. After spinning, the silk fibroin electrospinning membrane was peeled off from the release paper. Then, the silk membrane was soaked in an excess of 75% ethanol solution. After 2 h, the silk membrane was taken out carefully and placed in excess water to remove ethanol. The modified silk fibroin membrane was obtained by soaking in water three times (2 h for each time).

A total of 10 U/mL of the thrombin solution and 20 mg/ml of the fibrinogen solution were prepared. The two solutions were filtered and sterilized on an ultra-clean table, respectively, and then mixed at 4°C with a volume ratio of 1:3. Then, the mixture was coated on to the commercial polypropylene mesh. The modified silk fibroin membrane was adhered to one side of the polypropylene mesh and placed in a sterile incubator at 37°C (Forma Series II, Thermo Fisher, United States). After 20 min, the composited SFP mesh was obtained and stored in a sterile environment at 4°C for subsequent use. The average thickness of the silk fibroin membrane was 163.2 ± 35.5 μm, and the average thickness of the polypropylene mesh was 444 ± 4.9 μm. After the membrane was immersed in 75% ethanol, the structure of the fibers changed and became indistinguishable. Therefore, the average fiber diameter of the modified silk membrane was unable to be calculated.

### Structure analysis

Attenuated total reflection–Fourier transform infrared spectroscopy (ATR-FTIR, Nicolet iS20, Thermo Scientific, United States) was applied to characterize the structure of the original silk fibroin (SF) membrane and modified SF membrane. The original SF membrane was directly collected after the electrospinning process was finished. Then, the original SF membrane was immersed in 75% v/v aqueous ethanol for 2 h to get the modified SF membrane. Two groups of membranes were completely dried before the tests.

### *In vitro* cell viability characterization

A live/dead assay with fibroblast cells was used to investigate the viability and cell adhesion of the modified SF membranes. Briefly, the cells were cultured and proliferated in Dulbecco’s modified Eagle’s medium (DMEM, Gibco, United States) containing 10% FBS. The membranes were sterilized by being immersed in 75% aqueous ethanol and then cut into 5 mm × 5 mm rectangular shapes. After the membranes were immersed in the sterilized PBS solution five times to wash out residual ethanol, 500 μl of the cell suspension in a culture medium was added to the surface of the membranes at a density of 10^5^ cells/mL. The cells cultured only on TCPS culture plates were set as the blank group. The cells in all the groups were cultured for 24 h at 37°C in an incubator with 5% CO_2_. Calcein AM and PI were added according to the instructions to stain the live and dead cells, respectively. The stained cells were observed under a fluorescent microscope (AxioVert 200, Zeiss, Germany).

### Animal experiments

A total of 40 Sprague–Dawley male rats were divided into the SFP group and control group, with 20 rats in each group by the random number table method. The experimental animals were anesthetized with 3% pentobarbital sodium by intraperitoneal injection (30–40 mg/kg). All the implant materials were disinfected with ethylene oxide. The polypropylene mesh and SFP mesh were implanted in the groups, respectively (the silk fibroin surface was toward abdominal organs, and the polypropylene surface was toward the abdominal wall). The mesh size was 2 cm × 1 cm, and the mesh was fixed with a 5–0 Prolene suture. After the surgery, the experimental animals were placed at the appropriate temperature and humidity and reared in separate cages. The general conditions of the animals were observed. The rats were sacrificed by intraperitoneal injection of pentobarbital sodium overdose in batches on the 3rd, 7th, 14th, and 90th days after surgery. The observation contents were as follows:

1) Adhesion area on the mesh surface: according to Baptista et al. (2000), the mesh was visually divided into four equal quadrants for quantitating adhesions. Adhesions covering the mesh were estimated according to the area of attachment, ranging from 0% to 100% (no adhesions to completely covered). The intraabdominal structure involved, omentum, or intestine, and the surface appearance of the uninvolved mesh were noted and recorded.
2) Adhesion degree score of the mesh surface: according to LeBlanc et al. (2002), the implantation sites were exposed, and any adhesions observed between the implanted mesh and tissues were graded for extent and tenacity (0, indicating that no adhesions were present; 1, the adhesion was loose, and light blunt dissection was required for separation; 2, the adhesion was close, and heavy blunt separation was required for separation; 3, the adhesions requiring sharp dissection for separation; and 4, important organs adhering to the mesh surface, such as the intestine and liver).

### Histopathological examination

The whole layer of tissue except the skin was cut from the abdominal wall of the rat mesh implantation area, and pathological sections were stained with hematoxylin and eosin (HE). The whole layer of the abdominal wall except the skin was cut, and pathological sections were stained with hematoxylin and eosin. The inflammatory reaction in the implanted area and mesenchymal cells covering the surface of silk fibroin-adherent polypropylene material were observed. The hyperplasia of capillaries and adipocytes and the reaction of antigens were observed.

### Statistical analysis

The data were analyzed by SPSS 26.0 software (SPSS Inc., Chicago, IL, United States). Continuous variables were expressed as the mean ± standard deviation or median (interquartile), and a t-test or Mann–Whitney U test was performed. Categorical variables were analyzed by the Pearson chi-squared test or Fisher’s exact test. A *p*-value less than 0.05 was considered as a statistically significant difference for each statistical analysis.

## Results

### Structure analysis by ATR-FTIR

**FIGURE 2:**
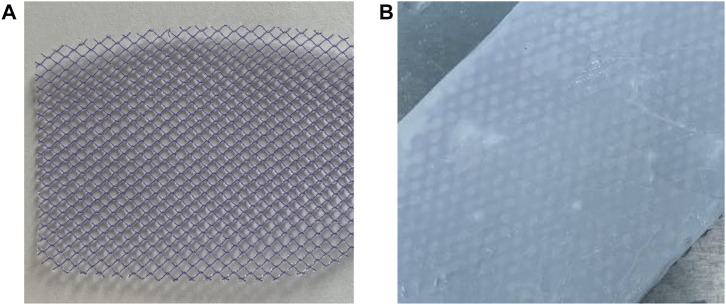
**(A)** Polypropylene mesh was used in the control group. **(B)** Silk fibroin and polypropylene composite biological mesh was used in the SFP group.

**FIGURE 3:**
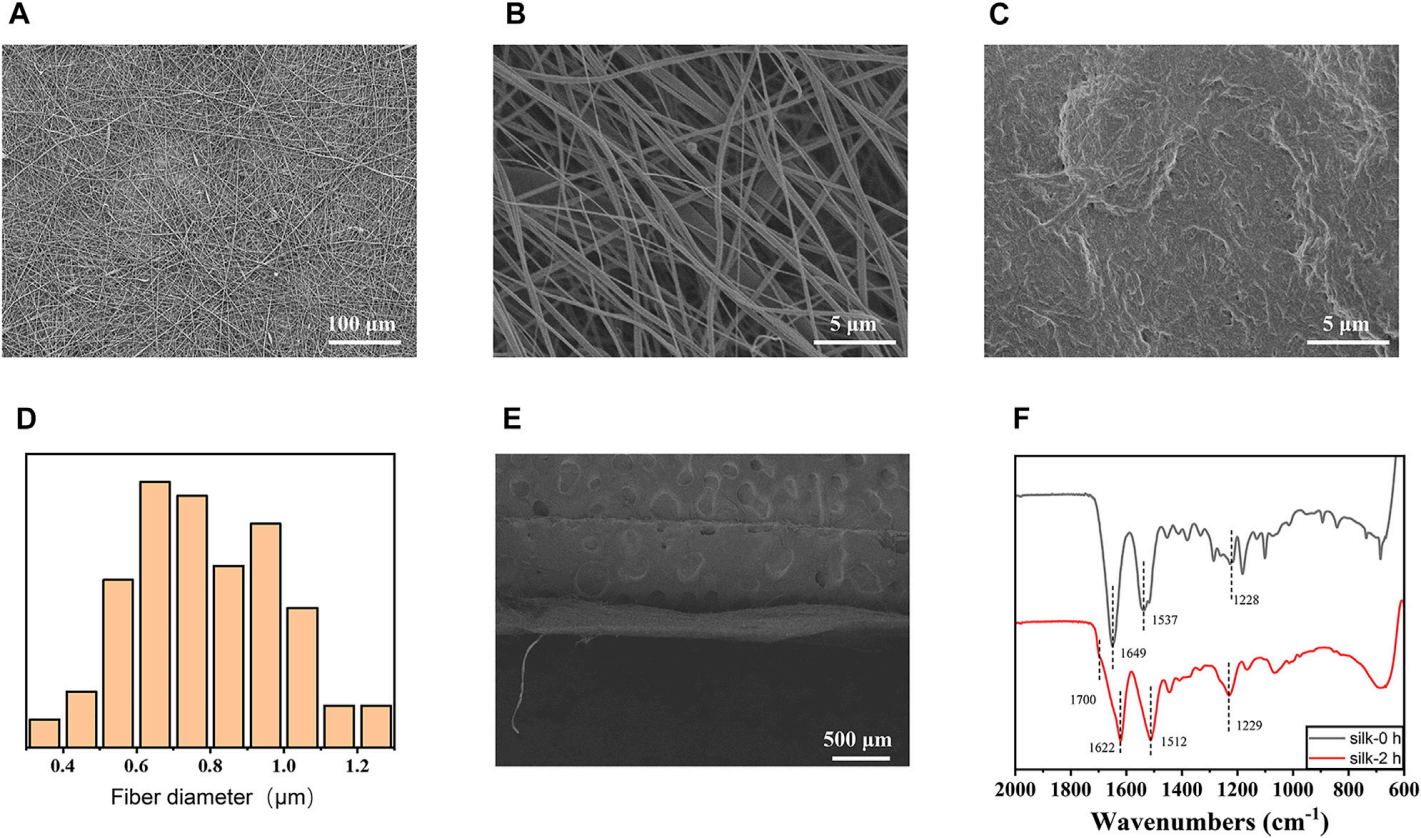
**(A)** Scanning electron microscopy (SEM) image of the electrospinning membrane before immersion in ethanol (×220). **(B)** SEM image of the electrospinning membrane before immersion in ethanol (×5000). **(C)** SEM images after immersion in ethanol (×5000). **(D)** Fiber diameter distribution statistics of the SFP mesh (*n* = 100). **(E)** Cross section of the silk fibroin membrane layer. **(F)** Infrared spectroscopy of the silk fibroin electrospinning membrane soaked in ethanol for 2 h shows that the β-folding component of silk fibroin was increased by ethanol aqueous solution treatment.

Figures 2A,B show the mesh products of the control group and the experimental group (SFP), respectively, for this study. In Figure 3, the appearance of peaks at 1600–1700, 1500–1600, and 1229 cm^−1^ were, respectively, referred to as amide I, II (β-sheet conformation), and amide III (random coil conformation), which are typical forms of peptides. After being immersed in 75% aqueous ethanol, the amide I band gradually shifted from 1655 to 1636 cm^−1^, and the amide II band shifted from 1543 to 1516 cm^−1^. It is well-known that hydrogen bonding can affect the peak position in the FTIR spectrum (Cezard et al., 2006). The shift of characteristic peaks in Figure 3F shows that a more stable structure was formed after the silk membrane was immersed in ethanol, which indicates that the random coil was transformed into β-sheet conformation (Min et al., 2004). Moreover, the weak peak at about 1700 cm^−1^ could be recognized as the antiparallel arrangement in the β-sheet domains of silk protein chains (Freddi et al., 1999; Nogueira et al., 2010).

### *In vitro* cell viability characterization

**FIGURE 4:**
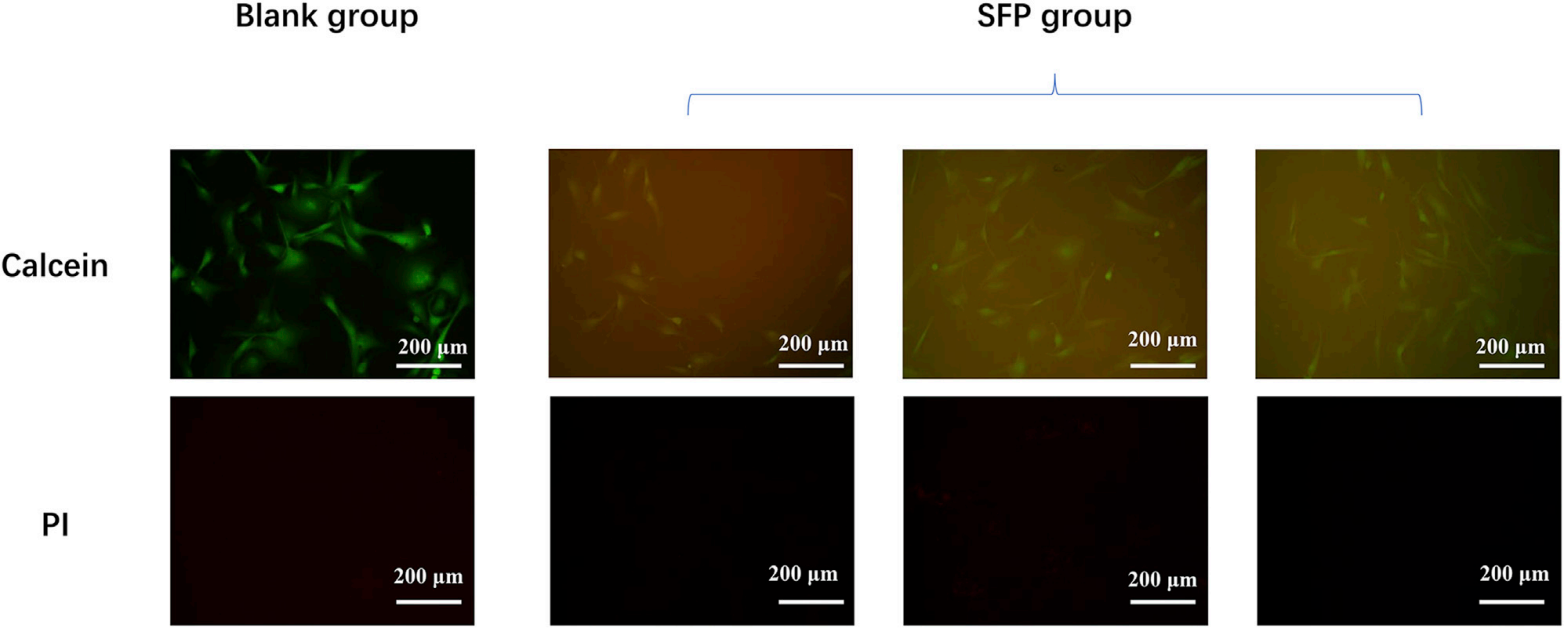
Experiment of the fibroblast culture on the modified silk fibroin membranes shows that the silk fibroin had a good biological activity.

Due to the limited light transmittance of the silk membrane, cells and fibrous membranes cannot be distinguished under bright fields. Figure 4 shows the photographs of cells in different groups being taken under the vision of a fluorescent microscope. The fluorescence could be excited by the presence of ethanol-treated SF membranes. Compared with the blank group, the fibroblasts also spread well when co-cultured with the membranes, and no dead cells could be observed. No significant difference in cell morphology between the two groups was found. It is believed that the ethanol-treated SF membranes have good cell viability.

### Animal experiments

The food intake of rats in the control group and SFP group basically returned to normal on the first postoperative day. In the control group, three rats died on the 2nd, 5th, and 10th days after surgery, respectively. The cause of death was investigated by laparotomy. It was found that the mesh surface of the dead rats was severely adherent on the 2nd and 5th days after surgery, which may lead to intestinal obstruction and death. The rats who died on the 10th day after surgery had intestinal tract adhesion to the mesh, and there was more fluid in the abdominal cavity, which was muddy in nature and accompanied by a foul odor. Therefore, the death of intestinal fistula infection was considered. Three rats in the experimental group died on the 2nd, 6th, and 18th days after surgery. The cause of death was investigated by laparotomy. It was found that adhesion on the mesh surface of dead rats on the 2nd and 6th days after surgery may cause intestinal obstruction and death. One rat died on the 18th day after surgery due to avoiding food intake. Abdominal exploration showed no obvious adhesion between the bowel and mesh in the rat.

**FIGURE 5:**
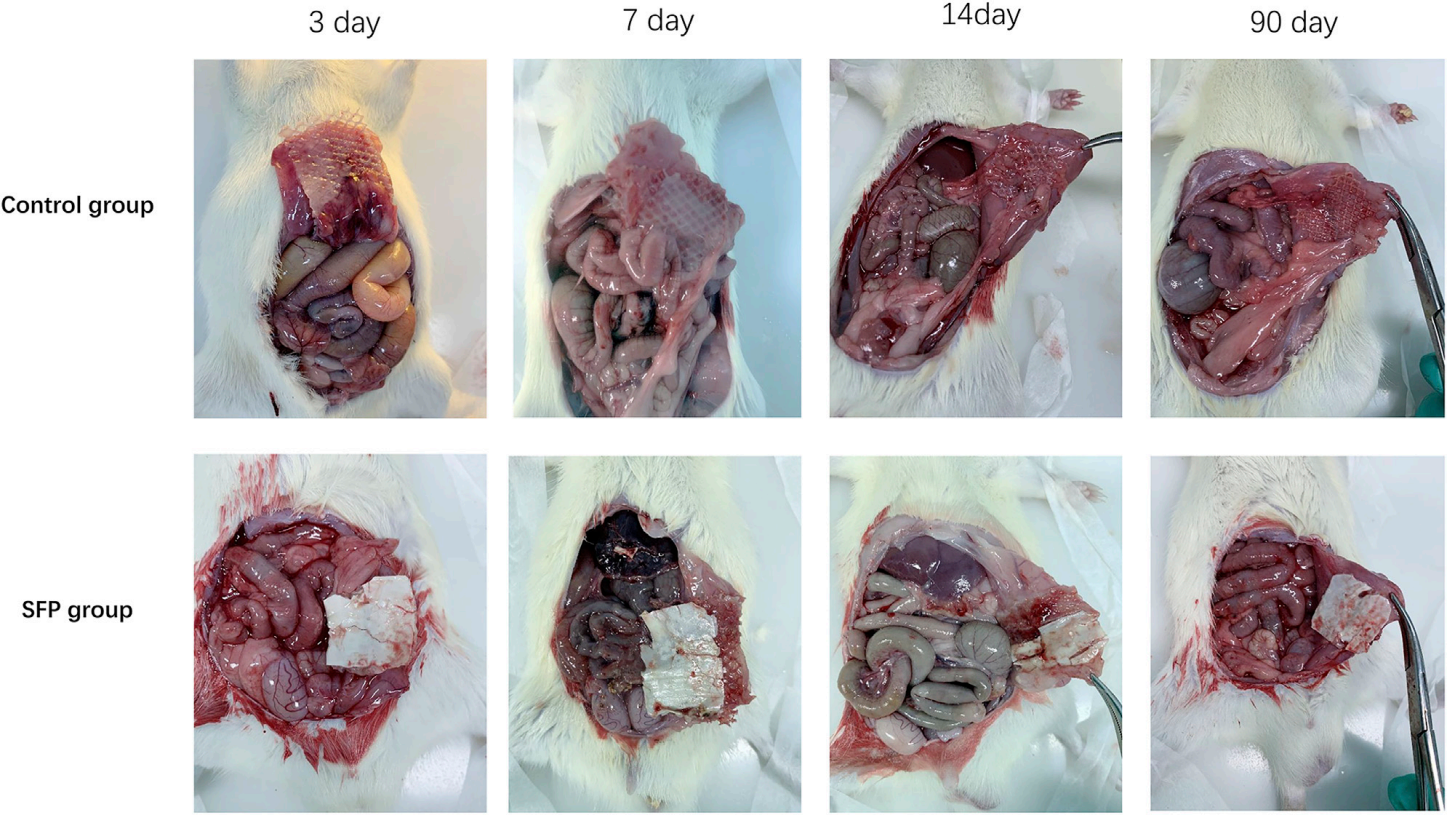
Comparison of intraperitoneal adhesions between two groups of rats on the 3rd, 7th, 14th, and 90th days after implantation of different meshes.

**FIGURE 6:**
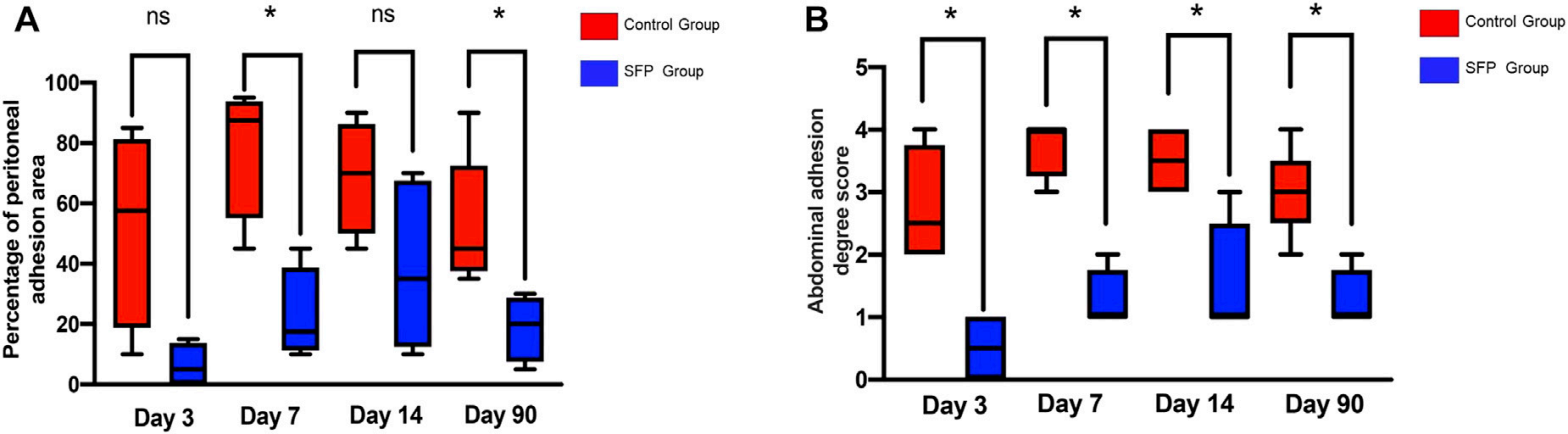
Valuation of the abdominal adhesion area **(A)** and adhesion degree score **(B)** after 3rd, 7th, 14th, and 90th days of different mesh implantation.(*p < 0.05, **p < 0.01, ***p < 0.001; ns, no significance).

The experimental animals in the two groups were sacrificed by peritoneal injection of pentobarbital on the 3rd, 7th, 14th, and 90th days postoperatively. The adhesion area and adhesion degree of the two groups were compared by laparotomy. The control group had different degrees of adhesion on the 3rd, 7th, 14th, and 90th days after surgery. However, there was almost no intraperitoneal adhesion on the 3rd and 7th days after surgery, and some rats only had mild adhesions on the 14th and 90th days after surgery in the SFP group. There were statistically significant differences in the postoperative intraperitoneal adhesion area and adhesion degree between the two groups (p < 0.05) (Figures 5, 6 and Table 1).

**TABLE 1:** Intraperitoneal adhesion of rats in the SFP group vs. control group.

Adhesion condition	Control group	SFP group	Z	*p*-value^#^
**Adhesion area (%)**	Median (interquartile)			
3 days	57.5 (27.5–77.5)	5.0 (0–12.5)	1.899	0.058
7 days	87.5 (65.0–92.5)	17.5 (12.5–32.5)	2.178	0.029
14 days	70.0 (55.0–82.5)	35.0 (15.0–65.0)	1.599	0.110
90 days	45.0 (40.0–55.0)	20.0 (10.0–27.5)	2.449	0.014
**Adhesion degree score**				
3 days	2.5 (2–3.5)	0.5 (0–1)	2.352	0.019
7 days	4 (3.5–4)	1 (1–1.5)	2.428	0.015
14 days	3.5 (3–4)	1 (1–2)	2.292	0.022
90 days	3 (3–3)	1 (1–1.5)	2.420	0.016

#Mann–Whitney U test

### Histopathological examination

**FIGURE 7:**
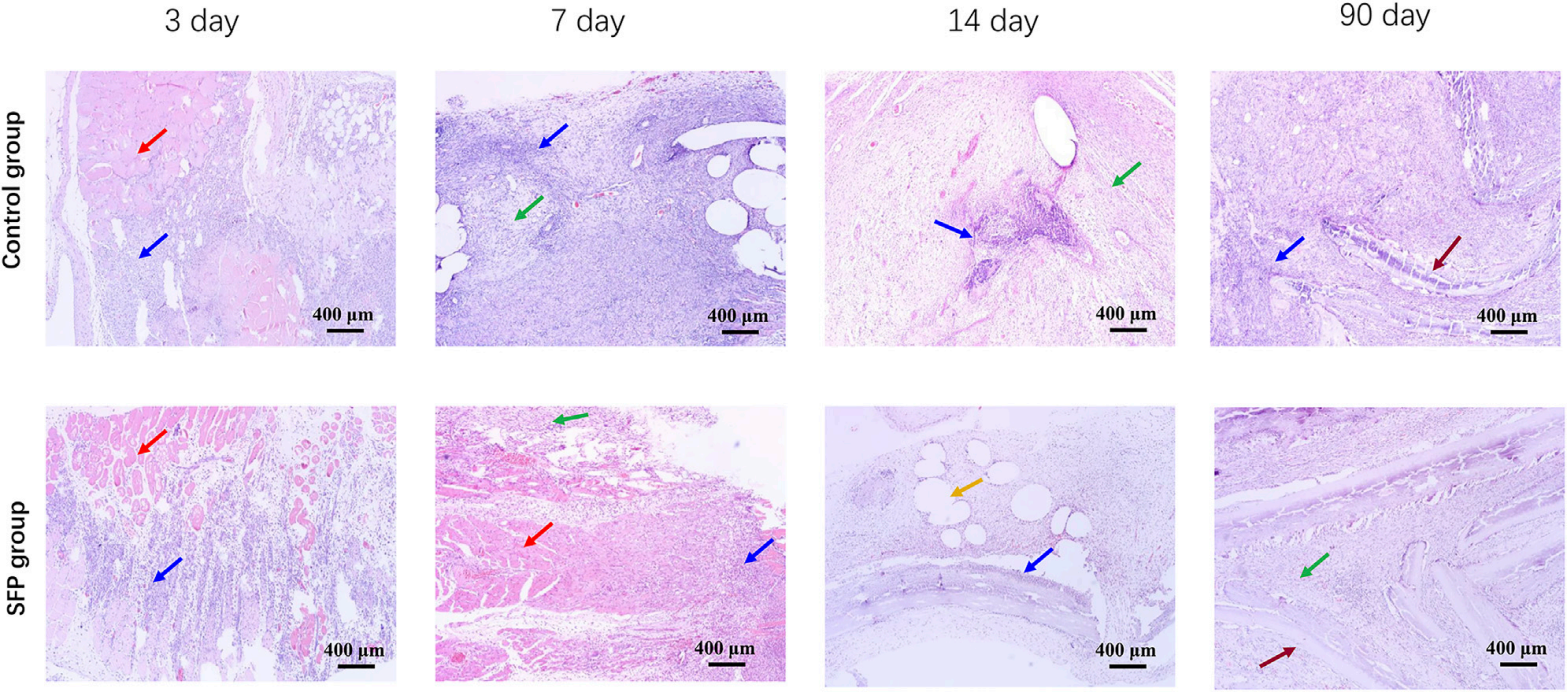
Histopathological results of abdominal inflammation in rats on the 3rd, 7th, 14th, and 90th days after implantation of different meshes. The red arrows show skeletal muscle tissue. The blue arrows show inflammatory cells. The green arrows show mesenchymal cells. The yellow arrows show adipocyte proliferation. The brown arrows show the mesh.

Histopathological examination confirmed that peripheral inflammation was severe in both the groups on the 3rd and 7th days postoperatively. On the 14th day after surgery, the inflammatory reaction around the SFP group was significantly reduced compared with the control group. The mesenchymal cells were well arranged and continuous, and there were more new capillaries and adipocyte proliferation under the mesenchymal cells. However, the inflammatory response of the SFP group basically disappeared on the 90th day, and the mesenchymal cells were in good shape and continuity. The silk fibroin material showed good biocompatibility (Figure 7).

## Discussion

Since the artificial mesh with a single component cannot meet the clinical requirements for incisional hernia repair of the abdominal wall, the composite mesh has made great progress and is mainly used to reduce the incidence and degree of adhesion after surgery so as to reduce the harm of adhesion complications. However, it has been reported recently that the composite mesh of synthetic materials may cause postoperative complications, such as intestinal obstruction, enterocutaneous fistulas, and pain (Yang et al., 2020b; Devin et al., 2021). Also, its degradation products may cause inflammation, erosion, and foreign body reactions. The composite mesh with faster absorption speed may aggravate the reaction degree of local tissues and accelerate the formation of postoperative adhesions.

Due to the occurrence of many clinical complications of the synthetic composite mesh, the application of biological meshes has been explored recently. The early biological mesh is formed by decellularization, disinfection, and sterilization of human cadavers or animal tissues. This kind of mesh has certain structural and functional proteins, which can be used as a scaffold to induce the cells and tissues of the defect after it is inserted into the body. Over time, the mesh can be completely absorbed and finally decomposed into substances needed by the body. However, there are also many shortcomings in the use of this biological mesh, such as its limited source, relatively high price, and the need to be soaked before use. The mesh size is also relatively small, and the incidence of complications is high, leading to certain limitations in clinical use. In recent years, the biological mesh used usually refers to a composite mesh made of natural polymer materials and other materials. The natural polymer materials are often used in the extracellular matrix such as collagen, elastin, and proteoglycan. Compared with other meshes, the composite biological mesh has the following advantages. 1) It can provide the required strength and degradation speed for the mesh. 2) It can significantly reduce inflammatory response. 3) It has strong anti-infection ability (Bellows et al., 2013; Cheng et al., 2014).

It was reported that an anti-adhesion layer could be formed by coating the surface of the polypropylene mesh with absorbable sodium hyaluronate, carboxymethyl cellulose, oxidized cellulose, and hyaluronic acid solution to prevent tissue adhesion. In our study, the SFP mesh was composed of polypropylene mesh and silk fibroin. In the surgical repair of abdominal incisional hernia, the side close to the abdominal wall is the polypropylene material, which is non-degradable and can provide sufficient tensile strength so as to achieve the purpose of repairing the defect through abdominal surgery and reducing the risk of recurrence of incisional hernia. The side facing the abdominal cavity is silk fibroin, which has good biocompatibility and can effectively adsorb peritoneal mesothelial cells and reduce the occurrence of postoperative abdominal adhesions. After a period of time, the silk fibroin is slowly degraded and metabolized in the body. In the process of degradation, it can promote the infiltration of host cells and produce collagen fibers, and induce fibroblasts to grow into the mesh pores, forming a “reinforced cement-like” structure. Thus, it can resist abdominal pressure, significantly reduce inflammation, and has a strong anti-infection ability. The SFP mesh will be permanently implanted in the body. It can significantly reduce the risk of abdominal adhesion and intestinal fistula.

We prepared a silk fibroin fiber membrane by the electrostatic spinning method, modified the silk fibroin fiber membrane by immersion in 75% ethanol, and then adhered it to the polypropylene mesh by fibrin hydrogel to prepare a new composite biological mesh. Structural analysis and cell viability tests *in vitro* showed that the SFP mesh had better cell viability than the polypropylene mesh. Animal experiments indicated that different degrees of adhesions appeared in the control group on the 3rd, 7th, 14th, and 90th days after surgery. In the SFP group, there was almost no intraperitoneal adhesion on the 3rd and 7th days after surgery, and only mild adhesions were found in some rats on the 14th and 90th days after surgery. There were statistically significant differences in the intraperitoneal adhesion area and adhesion degree between the two groups. Histopathological examination confirmed that the inflammation around rats in both the groups was serious on the 3rd and 7th days after surgery. On the 14th postoperative day, the inflammatory response in the SFP group was significantly less than that in the control group. Mesenchymal cells were arranged in a neat and continuous way, with more new capillaries and fat cell proliferation below the cells. However, the inflammatory response of the SFP group basically disappeared on the 90th day, and the mesenchymal cells were in good shape and continuity. The experimental results show that the silk fibroin composite biological mesh had good biocompatibility.

Our study suggests that this performance may be closely related to the biological, physical, and chemical properties of silk fibroin itself. First of all, the silk fibroin used was derived from the degluing of the natural cocoon silk. It is a natural biological macromolecule without physiological activity and contains 18 kinds of amino acids, such as alanine and serine. It has good biocompatibility, is non-toxic, non-irritating, biodegradable, and can be used as a cell scaffold for cell adhesion and growth. Second, through the histopathological examination of our samples, it was found that there were a large number of peritoneal mesenchymal cells on the surface of the silk fibroin in the experimental group. Some studies have shown that mesenchymal cells can prevent intraperitoneal adhesions in a variety of ways. Mesenchymal cells themselves can stimulate the proliferation and migration of other mesenchymal cells and secrete multifunctional cytokines, such as HGF, to stimulate the proliferation and migration of more mesenchymal cells. In addition, mesenchymal cells themselves can express transformed growth factor antibodies, which can promote the secretion of t-PA, inhibit the secretion of PAI-1, improve peritoneal fibrinolysis, and reduce the occurrence of postoperative abdominal adhesion.

However, there are still some limitations that need to be improved in our experiment. The sample size of the experimental animal is a little small. The adhesion degree between the silk fibroin and polypropylene mesh in the SFP mesh needs to be strengthened; otherwise, the silk fibroin may fall off from the polypropylene mesh. The strength of the silk fibroin itself also needs to be strengthened to avoid breaking. This may require an improvement in the fusion process of silk fibroin and polypropylene.

## Conclusion

Our findings confirmed that the silk fibroin and polypropylene composite biological mesh not only reduced the area and degree of adhesion in the abdominal cavity but also did not cause serious postoperative complications. The internal material is polypropylene, which can be used to increase the tension strength and reduce the risk of incisional hernia recurrence. The study indicates that the silk fibroin and polypropylene composite biological repair mesh will have a good application prospect in the field of abdominal wall hernia repair.

## Data Availability

The original contributions presented in the study are included in the article/Supplementary Material; further inquiries can be directed to the corresponding authors.
